# Handling benign interlobar lymphadenopathy during thoracoscopic lobectomy

**DOI:** 10.1111/1759-7714.13949

**Published:** 2021-04-03

**Authors:** Alfonso Fiorelli, Stefano Forte, Giovanni Natale, Mario Santini, Wentao Fang

**Affiliations:** ^1^ Department of Translation Medicine, Thoracic Surgery Unit Università della Campania “Luigi Vanvitelli” Naples Italy; ^2^ Istituto Oncologico del Mediterraneo (IOM) Viagrande Italy; ^3^ Department of Thoracic Surgery Shanghai Chest Hospital, Jiao Tong University Medical School Shanghai China

**Keywords:** lobectomy, lymphadenopathy, thoracoscopy

## Abstract

The presence of calcified or inflammatory lymph nodes between the target bronchus and pulmonary artery is a huge challenge when performing thoracoscopic lobectomy as it may frequently result in tearing of the vessel, and massive bleeding. Herein, we describe a simple strategy in which thoracoscopic lobectomy was safely completed in similar cases. After fissure dissection, the target pulmonary artery was exposed by more than two‐thirds of its circumference. A needle was passed across the nodes and the target vessel was closed with a proximal and distal suture. After dissection of lymphadenopathies, the target bronchus was exposed, and stapled. This strategy was applied with success to complete right lower lobectomies for cancer in three patients. No complications occurred during the operation. Only one patient had persistent air leaks that spontaneously ceased 11 days later. Final pathology showed pN0 disease in all cases.

## Graphical abstract

Calcified or inflammatory lymph nodes between the target bronchus and pulmonary artery is a huge challenge when performing thoracoscopic lobectomy. Here, we report a strategy to handle this clinical situation. A needle was passed across the nodes and the target vessel was closed with a proximal and distal suture, and then gradually transected with scissors. After dissection of adenopathies, the target bronchus was stapled.

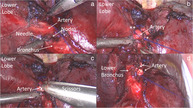

## INTRODUCTION

Since its introduction in the early 1990s, video‐assisted thoracoscopic surgical (VATS) lobectomy has become the strategy of choice for resection of early stage lung cancer. However, conversion to a thoracotomy occurred in up to 23% of previously reported cases,^1^, ^2^, ^3^, ^4^ and the presence of inflammatory or calcified lymph nodes (LNs) is one of the most common reasons for this. Herein, we describe a simple strategy to complete safely VATS lobectomy in the presence of lymphadenopathy between the target bronchus and pulmonary artery (PA).

## STUDY POPULATION

**FIGURE 1:**
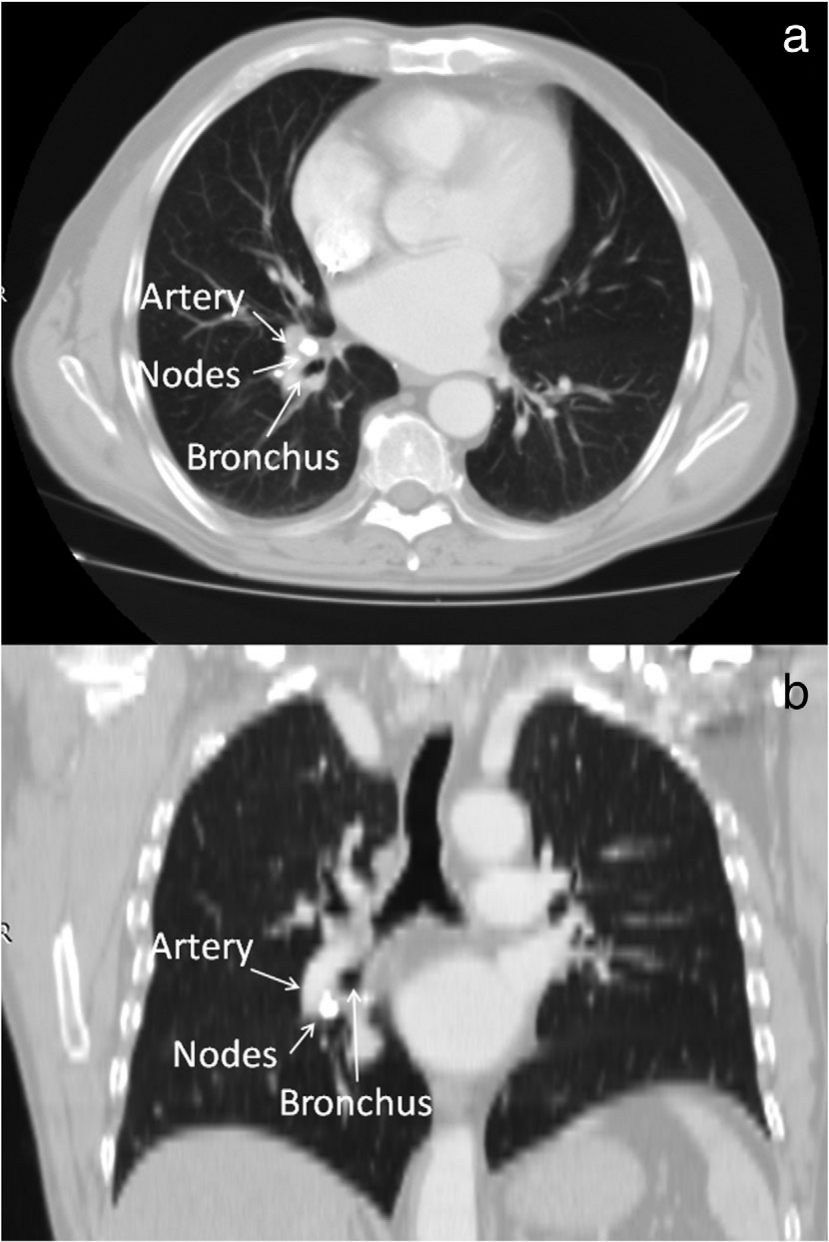
Computed tomography (CT) findings. (a) Axial view, and (b) coronal view showed calcified lymph nodes between lower pulmonary artery and lower bronchus (patient number 3)

This procedure was utilized in three patients (Table 1) to complete lower lobectomy where calcified or inflammatory LNs made the dissection of the lower PA from the target bronchus by conventional technique challenging. All patients had lung cancer at a clinically early stage, and did not receive induction therapy. Computed tomography (CT) findings (Figure 1) showed high‐density calcification in one case or nodular shadow with very close adherence to the lobar bronchus in the other two. The fat interspace between the lymphadenopathies, PA and bronchus was undetectable in all cases, and positron emission tomography (PET) scan indicated no signs of metastasis.

**TABLE 1:** Patient characteristics

Variables	Patient 1	Patient 2	Patient 3
Age	71	67	73
Sex	M	M	M
Comorbidity	COPD	COPD	COPD Tuberculosis Diabetes Vascular disease Cardiac disease
FEV1%	73%	77%	71%
Intervention	RLL	RLL	RLL
Histology	Squamous carcinoma	Adenocarcinoma	Adenocarcinoma
pStage	T1N0M0	T2N0M0	T3N0M0
Operative time (min)	125	120	355
Blood loss	200	150	550
Length of drainage (days)	5	4	11
Length of hospital stay (days)	6	5	13
Postoperative complications	None	None	Persistent air leaks

## TECHNIQUE

**FIGURE 2:**
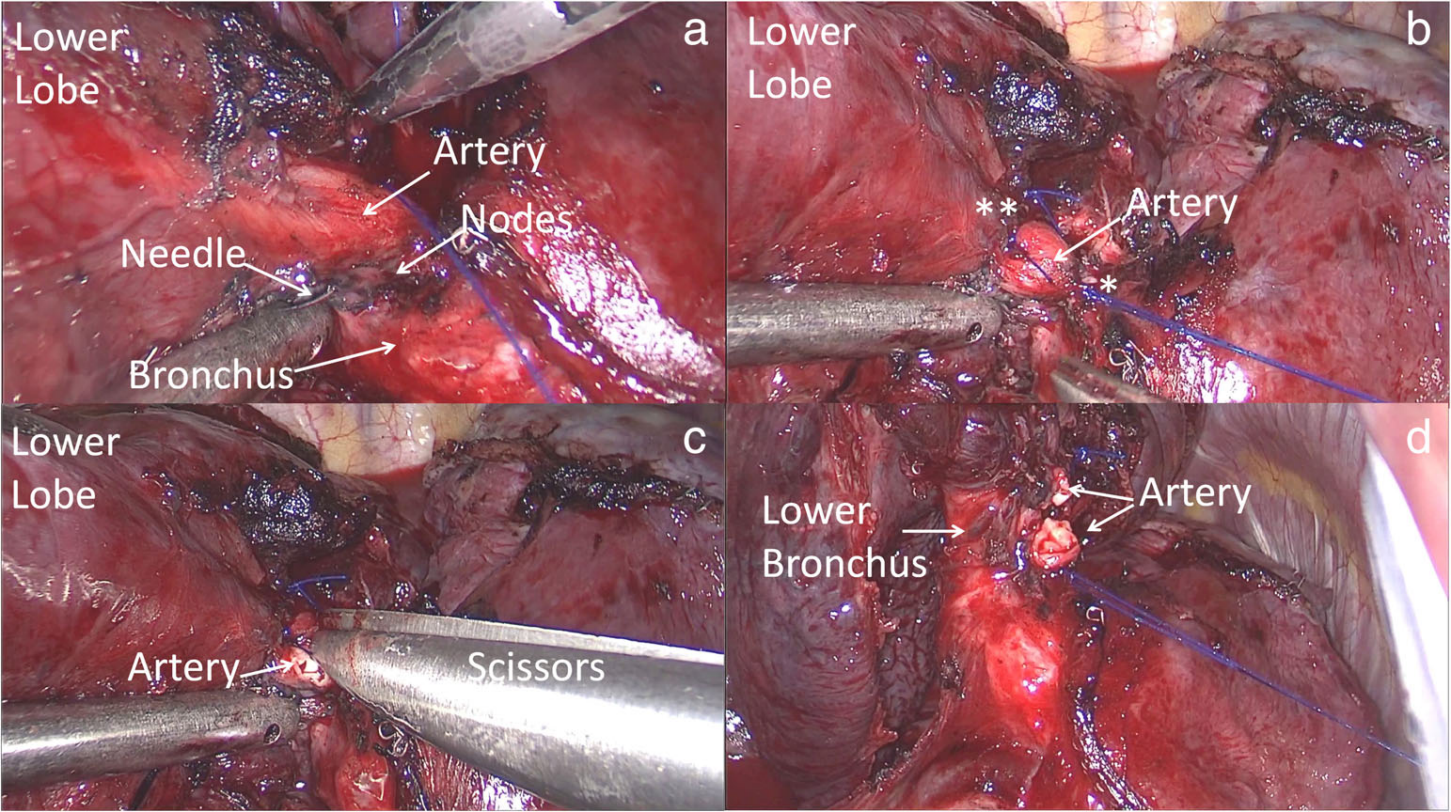
The inferior pulmonary artery and lower bronchus were frozen together by inflammatory lymph nodes. (a) A needle was passed across the nodes and (b) the target vessel closed with a proximal (*) and distal (**) suture. The vessel was then gradually transected with scissors (c). The lower bronchus was completely exposed (d), and stapled

Standard triportal VATS lobectomy was performed under general anesthesia and one‐lung ventilation in all cases. After resection of the pulmonary ligament, the inferior pulmonary vein was isolated. The fissure was then dissected and the inferior PA and target bronchus were found to be frozen together by inflammatory or calcified LNs. Intraoperative biopsy of nodes was negative for cancer, thus we proceeded to perform a lower lobectomy. First, the inferior pulmonary vein was stapled. The lower PA was then exposed by more than two thirds of its circumference. A needle (3‐0 prolene suture) was passed across the LNs and the suture was secured with a knot to close the vessel (Figure 1(a)). The procedure was performed on the proximal and distal sides of the target vessel (Figure 2(b)). Scissors were used to gradually transect the PA until the lower bronchus was completed exposed (Figure 2(c) and (d)). The lymphadenopathies were completely dissected from the bronchus via sharp dissection with scissors. The lower bronchus was then stapled in a standard manner. Finally, the operation was completed using standard surgical procedures, including radical LN dissection, leakage testing, intercostal nerve blockade, chest tube insertion and incision closure. The procedure is summarized in Video S1. No complications occurred during the operation. Only one patient had persistent air leaks that spontaneously ceased 11 days later. Final pathology showed pN0 disease in all cases.

## DISCUSSION

With recent developments in VATS technology and acquired experience, over time many clinical situations are not yet considered as contraindications for VATS lobectomy for experienced and skilled surgeons. By contrast, the presence of calcified or inflammatory LNs between the target bronchus and PA is still a huge challenge. The encircling of the bronchi or artery without any loose interspace may frequently result in tearing of the vessel and massive bleeding. The conversion rate in this clinical situation ranges from 9% to 41%, as previously reported.^1^, ^2^, ^3^, ^4^

To manage this issue, several techniques have been previously reported, but each of these present some disadvantages. Thus, the best strategy is still under debate. Some authors^5^, ^6^ have proposed that the main PA should be controlled in advance before dissecting the target vessel from the LNs and bronchus. A mechanical tear of the artery during dissection may then be repaired using 5‐0 nonabsorbable sutures. This technique requires additional mobilization of the main PA, and it is associated with an increase operative risk of vascular injury. Liu et al.^7^ stapled the target PA and bronchus together. Mechanical failure of the stapler and/or increased risk of bronchovascular or bronchopleural fistula are the main limitations.^1^ Furthermore, vessels, LNs and bronchus are stapled together, making the complete removal of adenopathies unfeasible. The procedure reported here overcomes all the above limitations. The target PA and bronchus are stapled separately facilitating complete LN resection and minimizing the risk of a fistula. Yet, the target vessel is first tied and then sectioned, preventing intraoperative bleeding.

We have the following suggestions for the success of the procedure. (i) Review of the preoperative chest CT scan helps to plan in advance how to perform the dissection, or to manage potential complications. Previous studies^8^, ^9^, ^10^ have evaluated the possibility of preoperatively predicting the presence of LN adhesions based on CT findings. A large dimension and high CT value of LN, presence of calcification,^8^ lack of a fat plane between the LN and the vessel,^9^ and high standardized uptake value (SUV) of LNs^10^ were all preoperative predictive factors of the presence of adhesions between LNs and the target vessel. (ii) During dissection, it is mandatory to expose the target PA by more than two thirds of its circumference in order to close the entire vessel with a proximal and distal suture as also reported in other series.^8^, ^11^ (iii) The needle should be passed across the nodes and not across the vessels to avoid vascular injury.

Our procedure is indicated in selected cases such as patients with calcified or inflammatory LNs between the target PA and the bronchial tree. In cases of hilar lymphadenopathies close to the main PA, more complex techniques such as vascular plastic resection and reconstruction should be used. Furthermore, when the target PA is not completely exposed, the target bronchus should be first transected in order to free the target PA, which is then stapled with the lymphadenopathies together. After removal of the specimen, the stump of the lobar bronchus is closed either with a stapler or absorbable suture.^11^

In conclusion, benign lymphadenopathy fused to vascular and bronchial structures should not be considered a main contraindication for VATS lobectomy. The present technique may be useful to handle this issue, minimizing the risk of intraoperative complications. Obviously, surgeons should be experienced in handling potential bleeding through a VATS approach and have the support of a team (assistant, anesthesiologist and nurses) ready to handle such situations. Finally, conversion to thoracotomy should always be a consideration.

## CONFLICT OF INTEREST

The authors disclose no conflict of interest.

## Supporting information


**Video S1** Video edited the main steps of the procedure as the transfixing suture across the node on the proximal or distal side of the target vessel; the ligation and transection of the target vessel and of the bronchus.
